# Drug Reprofiling to Identify Potential HIV-1 Protease Inhibitors

**DOI:** 10.3390/molecules28176330

**Published:** 2023-08-30

**Authors:** Sunday N. Okafor, Abigail Meyer, Jay Gadsden, Fadi Ahmed, Lilian Guzmán, Hashim Ahmed, José A. Fernández Romero, Pavimol Angsantikul

**Affiliations:** 1 Center for Biomedical Research, Population Council, New York, NY 10065, USA; sunday.okafor@unn.edu.ng (S.N.O.); hahmed@popcouncil.org (H.A.); jromero@popcouncil.org (J.A.F.R.); 2 Department of Pharmaceutical and Medicinal Chemistry, University of Nigeria, Nsukka 41001, Nigeria; 3 Department of Science, Borough of Manhattan Community College, The City University of New York, 199 Chambers St., New York, NY 10007, USA; fadi.a2016@gmail.com (F.A.); liliguzman0721@gmail.com (L.G.)

**Keywords:** HIV-1 protease, drug repurposing, drug discovery, HIV-1 protease inhibitors

## Abstract

The use of protease inhibitors in human immunodeficiency virus type 1 (HIV-1) treatment is limited by adverse effects, including metabolic complications. To address these challenges, efforts are underway in the pursuit of more potent and less toxic HIV-1 protease inhibitors. Repurposing existing drugs offers a promising avenue to expedite the drug discovery process, saving both time and costs compared to conventional de novo drug development. This study screened FDA-approved and investigational drugs in the DrugBank database for their potential as HIV-1 protease inhibitors. Molecular docking studies and cell-based assays, including anti-HIV-1 in vitro assays and XTT cell viability tests, were conducted to evaluate their efficacy. The study findings revealed that CBR003PS, an antibiotic currently in clinical use, and CBR013PS, an investigational drug for treating endometriosis and uterine fibroids, exhibited significant binding affinity to the HIV-1 protease with high stability. Their EC_50_ values, measured at 100% cell viability, were 9.4 nM and 36.6 nM, respectively. Furthermore, cell-based assays demonstrated that these two compounds showed promising results, with therapeutic indexes higher than 32. In summary, based on their favorable therapeutic indexes, CBR003PS and CBR013PS show potential for repurposing as HIV-1 protease inhibitors.

## 1. Introduction

To this day, acquired immunodeficiency syndrome (AIDS), caused by HIV-1, continues to pose a global health challenge. Despite the tremendous worldwide efforts to combat the disease, which led to a decline in its ranking from the 8th leading cause of global death in 2000 to the 19th in 2019, there were still 38.4 million people living with HIV in 2021. In low-income countries, HIV/AIDS remains among the top 10 leading causes of death [1]. To address this issue, scientists and relevant stakeholders such as the World Health Organization (WHO) are intensifying their strategies to combat this infectious disease. The WHO’s 2022–2030 global health sector strategy on HIV aims to reduce HIV infections from 1.5 million in 2020 to 335,000 by 2030 and decrease deaths from 680,000 in 2020 to under 240,000 in 2030 [2].

**Scheme 1:**
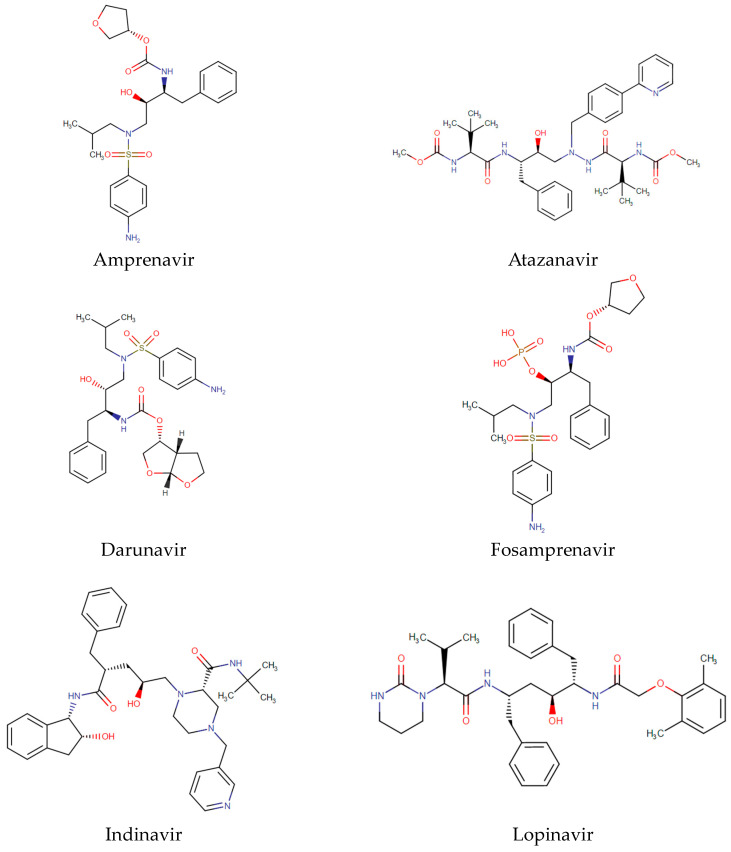

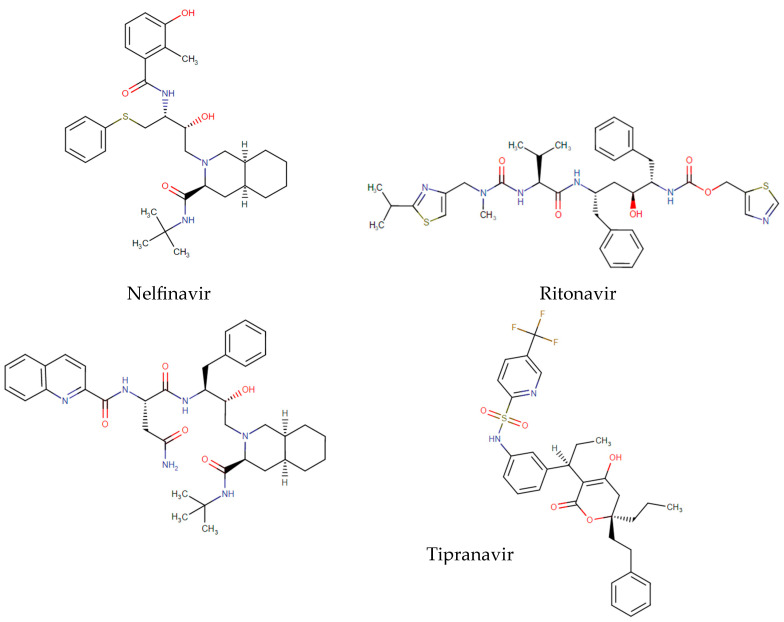
Chemical structures of HIV-1 protease inhibitors in clinical use.

Various classes of anti-HIV drugs are available, including binding inhibitors (such as CCR5 antagonists and post-attachment inhibitors), fusion inhibitors, reverse transcriptase inhibitors (including non-nucleoside reverse transcriptase inhibitors and nucleoside reverse transcriptase inhibitors), integrase inhibitors, and protease inhibitors. HIV-1 protease inhibitors stand out as one of the most potent reported anti-AIDS drugs to date and are essential components of highly active antiretroviral therapy (HAART) [3,4]. The current HIV-1 protease inhibitors on the market, depicted in Scheme 1, include saquinavir (SQV), indinavir (IDV), ritonavir (RTV), nelfinavir (NFV), amprenavir (APV), lopinavir (LPV), atazanavir (ATV), tipranavir (TPV), and darunavir (DRV). These inhibitors work by competitively binding to the active site of the HIV-1 protease enzyme. By doing so, they prevent the enzyme from cleaving viral polyproteins into functional units required for viral maturation and replication.

The conventional de novo drug discovery method is not only complex and laborious but is also a costly and high-risk process. Current reports estimate that the cost of bringing a drug candidate to the market is USD 1.8 billion [5]. Due to these inherent challenges, scientists are turning to computer-aided drug design (CADD) approaches. CADD encompasses two main strategies: structure-based drug design (SBDD) and ligand-based drug design (LBDD). SBDD has successfully contributed to the discovery of several drugs, including nine FDA-approved protease inhibitors in the past decade [6].

One valuable tool in modern drug discovery employed in CADD is drug repurposing (DR) (also known as drug repositioning). DR is a strategy aimed at identifying novel therapeutic agents from existing FDA-approved drugs in clinical use, as well as discovering new uses for approved or investigational drugs that extend beyond their original medical indications [7]. This emerging approach presents an effective means of discovering potential therapeutics for various diseases and infections. Furthermore, repurposed drugs may uncover new targets and pathways worthy of further exploration. To expedite the drug discovery process, drug repurposing is widely embraced, particularly in cases where drugs have already been proven safe and effective in humans and have received FDA approval for other indications [8].

Several repurposed drugs have been implemented to date. Antiviral drugs like remdesivir and favipiravir, as well as antimalarial drugs like Chloroquine and Hydroxychloroquine, are being investigated and repurposed for the treatment of COVID-19. Pfizer successfully repurposed Sildenafil, originally developed as an antihypertensive drug, for the treatment of erectile dysfunction. Thalidomide was repurposed for erythema nodosum leprosum (ENL) in 1964 and multiple myeloma in 1999 [9]. Raloxifene, initially indicated for osteoporosis treatment, has found valuable use in breast cancer treatment [10]. In 1989, Zidovudine, originally used in cancer treatment, was approved by the FDA as an anti-HIV drug [11].

The long-term use of protease inhibitors and other classes of antiretroviral therapy (ART) drugs is accompanied by myriad adverse effects. This is a rationale for exploring novel protease inhibitors, as the adverse effects contribute in part to low adherence to ART and subsequent therapeutic failure. The chronic use of protease inhibitors has led to metabolic complications related to glucose intolerance and overt type 2 diabetes [12,13,14]. These effects are believed to result from the inhibitory activity of protease inhibitors on IRS-1 phosphorylation, the association of phosphatidylinositol 3-kinase (PI3-kinase), and/or Thr^308^/Ser^473^-Akt, which subsequently block the translocation of GLUT4 vesicles to the plasma membrane [15,16]. Consequently, this inhibitory action leads to decreased glucose disposal in skeletal muscles and the development of insulin resistance [17,18]. Protease inhibitors have also been reported to induce lipolysis and lipid oxidation [19,20], elevate intramyocellular lipid accumulation, and impair adipocyte and glucose metabolism [21,22]. In addition to adverse metabolic effects, the emergence of multidrug-resistant (MDR) protease variants poses a serious threat to the efficacy of currently available protease inhibitors [23,24,25].

Thus, acknowledging the important role that protease inhibitors play in HIV treatment while considering the multitude of adverse effects associated with their prolonged use, our aim was to uncover novel protease inhibitors with improved therapeutic profiles and less toxic effects through a drug repurposing screen and subsequent toxicity analysis.

## 2. Results

### 2.1. Molecular Docking Study

**Figure 1:**
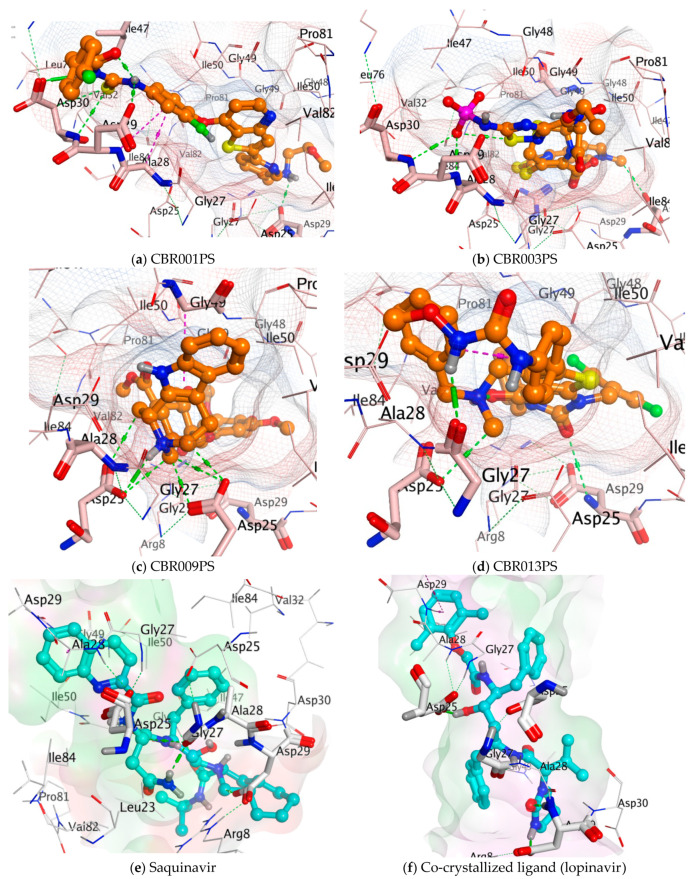
Binding poses of the drugs in the active binding site of HIV-1 protease.

Molecular docking studies generated several results, including the docking scores (using London dG and GBVI/WSA dG scoring functions) and chemical interactions of the drugs with the target, as presented in Table 1 and Table 2, respectively. Based on the high binding free energy and the in silico prediction of anti-HIV-1 activity (IC_50_ and percentage inhibition) shown in Table 1, the top four ranking drugs were selected for detailed molecular docking studies. Figure 1a–d illustrates the binding poses of these selected drugs in the active binding sites of HIV-1 protease. The docking protocol for this study has been outlined in Okafor et al., 2022 [26]. The drugs in Table 1 were selected after the molecular docking simulation of both FDA-approved and investigational drugs in the DrugBank database. The criteria for the final selection include the following: 1. Drugs with the least binding free energy, expressed in kcal/mol as measured via the scoring function GBVI/WSA dG, when compared to that of the standard drug (saquinavir); 2. Drugs for which the silico evaluation of anti-HIV-1 protease activity showed the least IC_50_ (µM) and a higher % inhibition compared to that of saquinavir. One notable finding from the docking study was that the selected drugs exhibited considerably lower binding free energy to the drug target compared to that of the known HIV-1 protease inhibitor saquinavir. The binding affinities of the top-ranking drugs ranged from −16.61 to −21.57 kcal/mol whereas saquinavir showed an affinity of −10.15 kcal/mol (Table 1). This difference could be attributed to the effective hydrogen bonding interactions between the ligand atoms of the drugs and the amino acid residues at the active binding site of HIV-1 protease.

**Figure 2:**
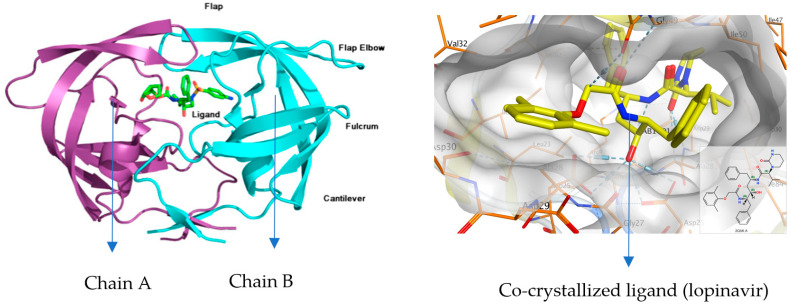
Crystal structure of HIV-1 protease target.

HIV-1 protease is characterized by having only one active-site Aspartic acid residue, whereas a catalytic diad requires two of such residues, suggesting that retroviral enzymes function as homodimers (Figure 2). Within the active binding site, several amino acid residues play important roles, including Gly^52^, Phe^53^, Ile^54^, Thr^80^, Pro^81^, Val^82^ and Ile^84^ [24]. Notably, certain residues such as Gly^27^, Asp^29^, Asp^30^, and Gly^48^ are relatively conserved as contact inhibitors. In our study, each drug interacted with at least four of these amino acids through diverse chemical interactions.

Table 2 provides the important protease–inhibitor hydrogen bonds observed in the crystal structures of CBR001PS, CBR003PS, CBR009PS and CBR013PS, including those mediated by water molecules. The table also shows the hydrogen bonding distances observed in these four crystal structures. Both CBR001PS and CBR013PS form hydrogen bonds with either of the two catalytic residues, Asp^25^ and Asp^25′^, in the floor of the active site, as well as other conserved amino acid residues (Gly^27^, Asp^29^, Asp^30^, and Gly^48^) within the active binding site of the protease. Detailed information regarding these specific chemical interactions can be found in Table 2.

**Figure 3:**
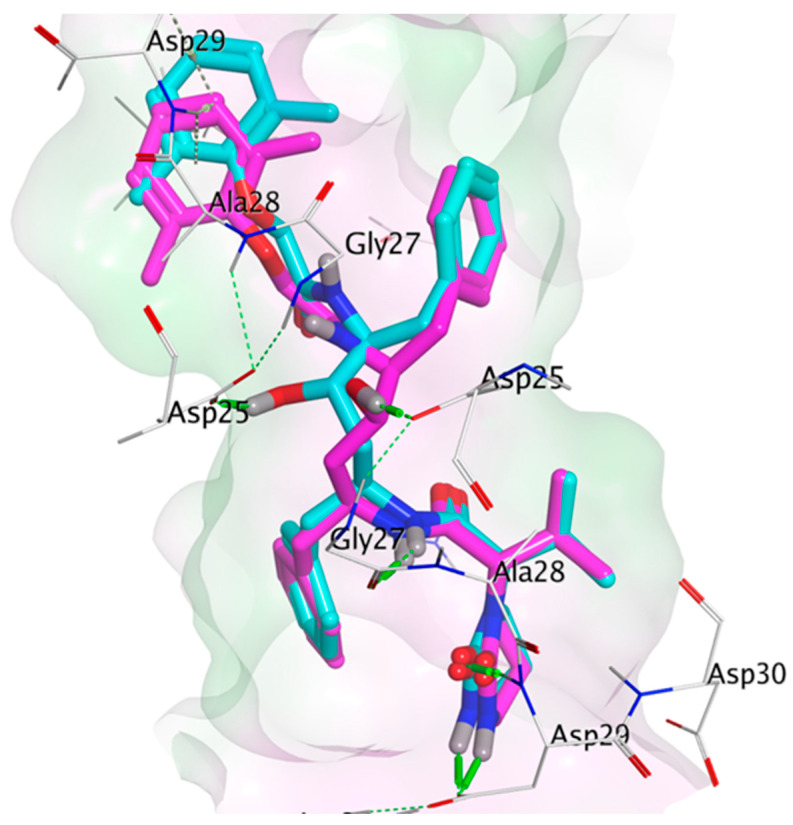
Validation of the docking protocol.

The co-crystallized ligand was retrieved and docked into its original binding pocket in the HIV protease. The docked ligand is shown in cyan, while the co-crystallized ligand is shown in purple with a root mean square deviation of 2.00 Å. Figure 3 shows the validation of the docking protocol.

### 2.2. In Vitro Anti-HIV-1 Assay

**Figure 4:**
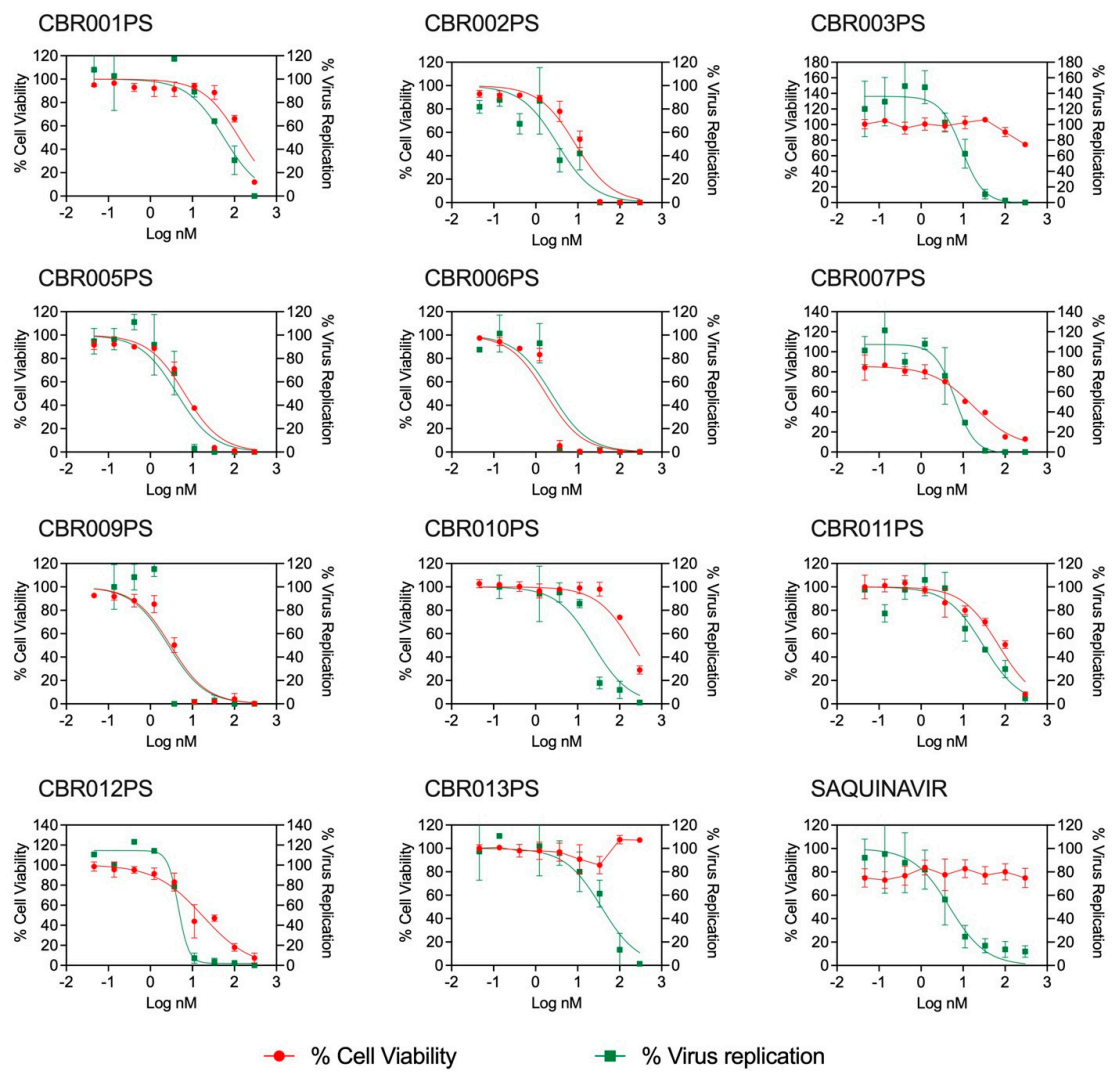
Analysis of selective antiviral activity of CBR003PS and CBR013PS in comparison to that of saquinavir. The panel shows the dose–response curves for cytotoxicity (regression lines represented in red) and antiviral activity (regression lines represented in green). The graph depicts the percentage of HIV-1_MN_ replication on the right y-axis and the percentage of cell viability on the left y-axis, both plotted against the logarithmic scale of drug concentration. Each data point represents the mean ± standard error (SE) of six replicates in two independent experiments.

HIV-1 protease plays a vital role in the HIV-1 life cycle. The enzyme is responsible for cleaving newly synthesized viral polyproteins, resulting in the production of mature proteins essential for the formation of infectious HIV-1 virions. The inhibition of the HIV-1 protease produces non-infectious HIV-1 progeny and limits viral spread. While the standard TZM-bl assay is widely used to screen the antiviral activity of antiviral molecules that interfere with HIV-1 replication, it measures the inhibition of HIV-1 replication as a function of the reduction in Tat-induced luciferase reporter gene expression after a single round of virus infection. This means that reporter gene expression occurs before the protease inhibitor can act at the end of the HIV-1 replication cycle. To overcome this limitation, the two-step procedure described in the methods section below was used to determine anti-HIV-1 activity. The results of the test are represented by the green regression lines in Figure 4, alongside the cytotoxicity assay results, shown in red, which were used to assess the selectivity of each drug tested. Among the drugs tested, compounds CBR003PS and CBR013PS exhibited the most promising selectivity when compared to the saquinavir control (saquinavir EC_50_ = 1.2 nM). Both CBR003PS (EC_50_ = 9.4 nM) and CBR013PS (EC_50_ = 36.6 nM) demonstrated EC_50_ values in the nanomolar range, with a therapeutic index exceeding 32 for CBR003PS. Similar to that of saquinavir, the CC_50_ values for both compounds were above 300 nM.

## 3. Discussion

By comparing in vitro to in silico results, a strong correlation was observed between the docking scores of the compounds and their in vitro anti-HIV-1 activities. For instance, in Table 1, CBR003PS exhibited predicted anti-HIV-1 activity with an IC_50_ value of 0.42 and a percentage inhibition of 58.78%. The molecular docking results presented in Table 1 and Table 2 demonstrate that potentially repurposed drugs mimic the mechanism of action observed in conventional protease inhibitors, as revealed by the molecular interactions with the target. CBR003PS and CBR013PS have similar molecular interactions with the co-crystallized ligands. They all formed strong H-bond interactions with the Asp^29^ of chain B. In addition, CBR013PS and the co-crystallized ligand interacted with the OD1 Asp^25^ of the chain B through a H-bond interaction.

The HIV protease belongs to the family of aspartic proteases and performs a vital function in HIV replication. The enzyme cleaves the Gag and Gag-Pol polyproteins generating mature infectious virions that can infect HIV target cells in the subsequent round of replication. Our cell-based method explores the inhibition of this maturation process in the CEM.SS cells and measures the presence of infectious virions in the TZM-bl assay. The selective index of each molecule is then calculated using the CC_50_/EC_50_ ratio that measures how selective the molecule is against the pathogen while causing minimal toxicity to the host cells. Based on this criterium, CBR003PS, CBR013PS, and saquinavir (our positive control) showed the highest ratio indicating better selectivity. Both CBR003PS and CBR013PS showed 100% viability in all the concentrations tested. CBR003PS, a parenteral cephalosporin, was initially developed to selectively target Gram-positive bacteria’s cell walls, which can explain the lack of toxicity in mammalian cells [27]. On the other hand, CBR013PS has shown a favorable safety profile in vitro and in vivo, which may also explain the lack of toxicity in our cell-based assays [28]. The favorable safety profile is an important point since it has been documented that conventional HIV protease inhibitors are associated with adverse side effects, especially after prolonged use. Some metabolic side effects have been reported, including glucose intolerance and clinically diagnosed type 2 diabetes [12,14]. A study by Nolan 2003 linked protease inhibitor therapy to insulin resistance and lipodystrophy [29]. These potentially repurposed drugs are void of the inherent adverse effects of conventional protease inhibitors.

Regarding the two lead compounds, CBR003PS and CBR013PS, CBR003PS, an antibiotic, shows promising selectivity as a potential HIV-1 protease inhibitor. However, it is crucial to highlight the risks associated with using an antibiotic long-term to treat HIV-1. Previous studies have explored the repurposing of antibiotics for diseases other than bacterial infections [30,31,32]. For instance, levofloxacin has recently been proposed as a potential drug to treat Alzheimer’s disease [32]. Nevertheless, considering the long-term use of medications associated with HIV-1 infection, it is essential to thoroughly assess the risks of antimicrobial resistance and the potential negative impacts the drug may have on the normal microbiome. On the other hand, CBR013PS presents intriguing potential as a candidate for HIV-1 treatment. As mentioned earlier, the drug has shown promise in the context of gynecological conditions. Further studies are warranted to explore the efficacy and safety of CBR013PS as a potential drug for HIV-1, offering a potential new approach to combat this persistent viral infection.

## 4. Materials and Methods

### 4.1. Molecular Docking

The crystal structure of the wild-type HIV-1 protease bound to lopinavir [6] was obtained from the RCSB Protein Data Bank (PDB ID: 2Q5K) (https://www.rcsb.org/structure/2Q5K, accessed on 22 August 2022). HIV-1 protease is recognized as a crucial therapeutic target for antiviral therapy in HIV/AIDS due to its vital role in the virus’ life cycle. It is responsible for processing the viral Gag and Gag-Pol polyproteins into essential structural and functional proteins required for viral maturation. Consequently, inhibiting this function results in the production of immature and non-infective HIV. HIV-1 protease, classified as an aspartyl protease, exists as a homodimer with each monomer containing 99 residues (Figure 2). The active site is situated at the core of the dimerization interface, housing the catalytic triad composed of Asp^25^, Asp^29^ and Gly^27^.

This crystallographic structure of the wild-type HIV-1 protease receptor was prepared using the Quickprep module within the Molecular Operating Environment (MOE) 2022 software. The protein underwent energy minimization utilizing the Amber12:EHT force field and the reaction field solvation model [31]. Refinement was performed along a root mean square (RMS) gradient of 0.05 kcal/mol/Å^2^. All computational procedures were carried out using the MOE. The docking protocols have been thoroughly validated in Okafor et al., 2022 [26]. Figure 3 shows the validation of the docking protocols.

### 4.2. In Silico Anti-HIV Activity Prediction

The web-based algorithm HIVProtI was employed for the in silico prediction and design of protein-specific anti-HIV-1 compounds (http://bioinfo.imtech.res.in/manojk/hivProti; accessed on 1 November 2022). The tool was developed by experimentally evaluating the IC_50_ and percentage inhibition activity of diverse inhibitor datasets against three HIV proteins, namely reverse transcriptase RT, protease, PR, and integrase, IN [33].

### 4.3. In Vitro Anti-HIV Assays to Measure Selective Activity

#### 4.3.1. Cells and Virus

CEM.SS and TZM-bl cell lines were obtained from the NIH AIDS Reagent Program (Germantown, MD, USA). CEM.SS cells were cultured in RPMI 1640 medium (Thermofisher Scientific, Waltham, MA, USA) supplemented with 10% heat-inactivated fetal bovine serum (FBS; Thermofisher Scientific), 50 U/mL of penicillin, and 50 μg/mL of streptomycin (Thermofisher Scientific). TZM-bl cells were grown in Dulbecco-modified Eagle medium (DMEM; Thermofisher Scientific), supplemented with 10% FBS and with 50 U/mL of penicillin and 50 μg/mL of streptomycin. The HIV-1_MN_ laboratory strain was provided by Jeffrey D. Lifson (Leidos Biomedical Research, Inc., Frederick, MD, USA).

#### 4.3.2. Cytotoxicity

Clear 96-well U-bottom plates were used for the cytotoxicity assay. An amount of 100 μL of CEM.SS cells were added to each well at a concentration of 1 × 10^5^ cells/mL. Briefly, 100 μL of each compound dilution at 2× was added in triplicate. Cell controls received 100 μL of complete medium (RPMI 1640 medium supplemented with 10% FBS and with 50 U/mL of penicillin and 50 μg/mL of streptomycin). Nine decimal dilutions of each compound were tested in complete medium. The plates were incubated at 37 °C, 5% CO_2_, and 98% humidity for 72 h. After 72 h of incubation, the XTT assay was performed to estimate cell viability using the procedure previously described [34]. Absorbance measurements were obtained using a Molecular Device Spectramax iD3 microplate reader and the SoftMax Pro software 5.4.5 (Molecular Devices, San Jose, CA, USA).

#### 4.3.3. Anti-HIV-1 Activity

A two-step procedure was employed to assess anti-HIV-1 activity. In the first step, CEM.SS cells were infected in the presence of different drug concentrations (as listed in Table 1). The infectivity of the virus recovered from the cell supernatants was then evaluated in a second round using the TZM-bl assay.

1. CEM.SS cells were seeded in clear 96-well U-bottom plates (Thermofisher Scientific) following the procedure described for the cytotoxicity assay. Triplicate wells received 50 μL of each compound dilution at 4X. Virus and cell control triplicates received 50 μL and 100 μL of complete medium (RPMI 1640 medium supplemented with 10% FBS and with 50 U/mL of penicillin and 50 μg/mL of streptomycin), respectively. The same nine concentrations of each compound tested in the cytotoxicity assay were applied in the antiviral assay, with all dilutions performed in complete medium. Briefly 50 μL of HIV-1_MN_ was added to all the wells except for cell controls at a concentration of 200 infectious particles per well. The plates were incubated at 37 °C, 5% CO_2_, and 98% humidity for 72 h.
2. Briefly, 24 h prior to completing the incubation of step 1, white opaque 96-well flat-bottom plates were seeded with TZM-bl cells at a concentration of 1 × 10^5^ cells/mL (100 μL per well) in complete medium. The plates were incubated at 37 °C, 5% CO_2_, and 98% humidity overnight. On the following day, 100 μL of the supernatant from each well was carefully transferred to the TZM-bl plates. The cells were spinoculated by spinning down the plates at 1740 g, for 1 h and 40 min, at 23 °C in a Sigma 4-16K centrifuge using a plate rotor at 2 × 96 (Qiagen, Hilden, Germany). After spinoculation, 100 μL of complete fresh medium was added to all wells. The plates were incubated at 37 °C, 5% CO_2_, and 98% humidity for 72 h and then stained by performing the multinuclear-activated galactosidase indicator (MAGI) assay as previously described [34]. The number of infected cells per well was estimated using C.T.L. ImmunoSpot (Cellular Technology Ltd., Shaker Heights, OH, USA).

### 4.4. Data Analysis

The XTT data obtained from the SoftMax Pro software 5.4.5 or the MAGI data from the C.T.L. ImmunoSpot were manually entered and analyzed by using Microsoft Excel 16.76 and GraphPad Prism Software 9.4.0, Inc. (San Diego, CA, USA) GraphPad Prism Software was used for curve-fitting analysis and calculating the half-maximal cytotoxic concentration (CC_50_) or half-maximal effective concentration (EC_50_) of all compounds. To estimate selectivity, the therapeutic index was calculated by comparing the CC_50_/EC_50_ ratios.

## 5. Conclusions

The high-throughput screening of FDA-approved and investigational drugs has yielded two promising drug candidates with significant binding affinity with the HIV protease. CBR003PS and CBR013PS demonstrated EC_50_ values of 9.4 nM and 36.6 nM, respectively, at 100% cell viability. Cell-based assays identified these two compounds as the most promising, with therapeutic indexes exceeding 32. Therefore, the antibiotics CBR003PS, currently in clinical use, and CBR013PS, an investigational drug for treating endometriosis and uterine fibroids, show potential for repurposing as HIV-1 protease inhibitors.

## Figures and Tables

**Table 1:** Binding free energy, ΔG (kcal/mol), and in silico (HIVProtI)-predicted anti-HIV protease activities of potential drugs for repurposing.

S/N	Drug	Current Indication	Docking Scores (kcal/mol)		Anti-HIV Protease Activity ^c^	
			GBVI/WSA dG ^b^	London dG	IC_50_^d^ (µM)	% Inhibition
1	Saquinavir	HIV-1 protease inhibitor	−10.15	−7.81	4.58	58.4
2	CBR001PS	Thrombin inhibitor	−16.75	−7.70	20.68	62.43
3	CBR002PS ^a^	Antiviral—Hepatitis C Virus	−17.35	−6.31	−4.19 ^e^	52.52
4	CBR003PS	Antibiotic	−19.04	−8.73	0.42	58.74
5	CBR004PS	Antibiotic	−21.57	−9.20	−9.05 ^e^	59.05
6	CBR005PS	Vitamin	−17.61	−9.17	23.61	53.96
7	CBR006PS ^a^	Treatment of atherosclerosis	−15.05	−8.96	0.03	52.04
8	CBR007PS	Anti-hypertension	−16.12	−8.37	0.04	51.89
9	CBR008PS	Treatment of neurogenic bladder dysfunction or myasthenia gravis	−14.47	−8.72	0.01	61.89
10	CBR009PS ^a^	Anti-cancer	−19.32	−8.84	23.38	60.97
11	CBR010PS	Diabetes mellitus, Type 2	−16.89	−9.88	−81.75 ^e^	54.08
12	CBR011PS	Antihypertensive drug (ACEI)	−15.22	−8.78	5.43	56.75
13	CBR012PS	ACEI	−17.28	−8.08	0.04	52.48
14	CBR013PS ^a^	Treatment in endometriosis and uterine fibroids	−16.61	−10.51	−10.75 ^e^	60.55

^a^ Investigational drug; ^b^ GBVI/WSA dG; ^c^ in silico-predicted using HIVProtI; ^d^ IC_50_: half maximal inhibitory concentration. ^e^ Negative values probably indicating the least possible IC_50_ values that can be obtained from the algorithm.

**Table 2:** Chemical interactions with amino acid residues in the active binding site of HIV-1 protease.

Drug	Ligand	Receptor ^a^	Interaction	Distance (Å)	Energy (kcal/mol)
**CBR001PS**	N 8	O Gly^48^ (B)	H-donor	3.04	−1.5
	O 3	O HOH 226 (A)	H-acceptor	2.75	−3.6
	O 4	CB Pro^81^ (A)	H-acceptor	3.39	−0.3
	N 12	OD2 Asp^25^ (A)	Ionic	3.38	−0.8
	N 14	OD1 Asp^25^ (A)	Ionic	3.93	−0.6
	N 14	OD1 Asp^25^ (A)	Ionic	3.07	−4.0
	6-ring	CA Ala^28^ (B)	pi-H	4.25	−0.2
	6-ring	N Asp^29^ (B)	pi-H	3.65	−0.9
	6-ring	N Asp^30^ (B)	pi-H	4.39	−0.6
**CBR003PS**	C 53	OD2 Asp^30^ (A)	H-donor	3.12	−0.8
	O 8	CA Ala^28^ (A)	H-acceptor	3.11	−0.5
	O 13	N Asp^29^ (B)	H-acceptor	3.06	−1.6
	O 13	N Asp^30^ (B)	H-acceptor	3.51	−2.0
**CBR009PS**	N 9	OD2 Asp^29^ (A)	H-donor	3.01	−0.7
	N 14	OD2 Asp^29^ (B)	H-donor	3.18	−1.4
	N 16	OD2 Asp^30^ (B)	H-donor	3.17	−5.0
	S 2	N Asp^30^ (B)	H-acceptor	3.71	−0.8
	S 2	CB Asp^30^ (B)	H-acceptor	3.52	−0.5
	N 9	OD2 Asp^29^ (A)	Ionic	3.01	−4.4
	6-ring	CA Ala^28^ (B)	pi-H	4.15	−0.9
	6-ring	N Asp^29^ (B)	pi-H	4.43	−0.5
**CBR013PS**	C 19	OD1 Asp^25^ (B)	H-donor	3.30	−1.3
	O 6	N Asp^30^ (A)	H-acceptor	3.32	−0.8
	6-ring	N Asp^29^ (B)	pi-H	3.75	−0.6
	6-ring	N Asp^30^ (B)	pi-H	4.12	−0.5
**Saquinavir**	N 11	O GLY^27^ (A)	H-donor	3.02	−2.3
	C 16	OD2 ASP^29^ (A)	H-donor	3.27	−1.1
	C 33	O GLY^27^ (A)	H-donor	3.41	−0.7
	O 1	N GLY^48^ (A)	H-acceptor	3.01	−2.5
	6-ring	CA ALA^28^ (B)	pi-H	3.97	−0.9
**Co-crystallized ligand**	N 2	OD2 ASP^29^ (A)	H-donor	2.87	−3.7
	N 3	O GLY^27^ (A)	H-donor	3.16	−1.4
	O 4	OD1 ASP^25^ (B)	H-donor	2.70	−3.6
	O 1	N ASP^29^ (A)	H-acceptor	3.02	−3.0
	6-ring	N ASP^29^ (B)	pi-H	3.93	−1.1
	6-ring	CB ASP^29^ (B)	pi-H	4.06	−0.6

^a^ (A) represents interactions with protein chain A and (B) represents interactions with protein chain B.

## Data Availability

The data presented in this study are available on request from the corresponding author. The data are not publicly available due to privacy restrictions.
